# Editorial: Alternative and expanding views on central respiratory chemoreception in health and disease

**DOI:** 10.3389/fphys.2024.1403768

**Published:** 2024-04-05

**Authors:** Jaime Eugenín, George B. Richerson

**Affiliations:** ^1^ Departamento de Biología, Facultad de Química y Biología, Universidad de Santiago de Chile, USACH, Santiago, Chile; ^2^ Departments of Neurology and Molecular Physiology and Biophysics, University of Iowa, Iowa City, IA, United States

**Keywords:** central chemoreception, estrogen, panic disorder, hypercapnia, taupathy, alzheimer disease, reactive microglia, hypoventilation syndromes

Central respiratory chemoreception is a fundamental brain function for adjusting breathing to physiological demands and for systemic CO_2_ and H^+^ homeostasis. Hence, failure in central chemoreception is a known feature of life-threatening human conditions such as Congenital Central Hypoventilation Syndrome (CCHS), Sudden Infant Death Syndrome (SIDS), and Rett Syndrome (RS) (Feldman et al., 2003; Kinney et al., 2009; Nattie and Li, 2012; Guyenet and Bayliss, 2022).

Several decades ago, pioneering work established that the ventral medullary surface contains three regions showing chemosensitivity to topical acidification, which were implied in the control of breathing (Severinghaus, 1998). These areas were named: M for the rostral area (after Robert Mitchell), S for the intermediate area (after Marianne Schläfke) and L for the caudal area (after Hans Loeschcke) (Severinghaus, 1998). In later studies, it was shown that acidification of other brainstem areas also triggered hyperventilation, suggesting that central respiratory chemoreceptors are widely distributed along the brain stem (Coates et al., 1993; Feldman et al., 2003; Nattie and Li, 2012). It was once believed central chemoreception involves only a limited number of specialized neurons in specific regions, but recently more varied hypotheses have been proposed. Thus, much of the recent experimental effort has been devoted to determining the type of cell and the molecular mechanisms that endows these cells with chemosensitivity. Although not exempt of controversies and, sometimes, in absence of definitive evidence, it seems that neurons and astrocytes play a crucial role as central chemoreceptors (Richerson et al.; Gourine et al., 2010; Beltran-Castillo et al.; Guyenet and Bayliss, 2022)

That a type of cell is a true respiratory chemoreceptor, implies that this cell intrinsically senses hypercapnia/acidosis and, because of this, activates the respiratory network. Accordingly, a set of five experimental criteria have been proposed to be necessary and sufficient for a particular type of cell to be a central respiratory chemoreceptor (Guyenet and Bayliss, 2022). These criteria are: “*1) Cell-specific activation and inhibition have opposite effects on breathing; 2) Cell-specific inhibition blunts effects of CO*_*2*_ *on breathing; 3) Cell activity is CO*_*2*_ */H* ^*+*^ *-modulated in vivo; 4) Cell modulation by CO*_*2*_ */H* ^*+*^ *is at least partly a direct effect; 5) Disrupting the mechanism for direct CO*_*2*_ */H* ^*+*^ *sensing selectively in the presumptive chemoreceptor cell interferes with CO*_*2*_ *-stimulated breathing*” (Guyenet and Bayliss, 2022).

In the minireview by Gonye and Bayliss, the authors analyze the available evidence for retrotrapezoid nucleus (RTN), serotonergic raphe nucleus (RN), medullary astrocytes, locus coeruleus, and lateral hypothalamus to satisfy the five criteria for chemoreceptors. This is an interesting work that revealed that in all of the cases, despite great advances in their study, evidence is still lacking to assure unequivocally that each of these types of cell are true chemoreceptors. Although the criteria are reasonable and exacting, they are linked to the assumption that respiratory chemoreception resides on a single type of cell. Whether central respiratory chemoreception in a particular brainstem area emerges from interactions between different types of cells is an open question. For example, recent work suggests that serotonergic raphe neurons are essential for expression of chemosensory responses of other chemosensory neurons, like those in the RTN (Wu et al., 2019). Other chemoreceptor neurons may depend on interaction with astrocytes to fully express chemosensitivity.

Nowadays it is clear that central CO_2_/H^+^ -sensitive areas are not restricted to the brainstem (Nattie and Li, 2012; Vollmer et al., 2016). In fact, in the hypothalamus, one can find CO_2_-sensitive neurons that have been associated with panic disorder (Nattie and Li, 2012; Vollmer et al., 2016). As part of this Topic Research, Kinkead et al. reviewed how panic disorder can disrupt respiratory control and how stress, biological sex, and neuroendocrine mechanisms influence respiratory control underlying pathophysiology of panic disorder. This minireview implicitly embraces the idea that the function of structures generating central chemoreception also depends on and is modulated by other systems, like the neuroendocrine system. Furthermore, ventilatory effects and fear and escape behaviors seem intimately connected, since despite CO_2_ being able to increase ventilation at a lower threshold than panic associated-behaviors, in patients with panic disorder, CO_2_-thresholds for inducing hyperventilation, fear, and escape are lower, and the intensity of reactions is greater than that observed in controls.

The Topic Research also includes two experimental articles. The manuscript by Marciante et al. shows that during presumptive sleep, rTg4510 mice, that develop tauopathy initially in the hippocampus and cortex which then progresses to the brainstem, show disruption of the respiratory rhythm (sighs and apneas) and blunting of O_2_- and CO_2_-chemoreflexes. Breathing dysregulation was observed even before neurodegeneration was declared and the severity of breathing irregularity and impaired ventilatory chemoreflexes were age-dependent and strongly associated with the accumulation of hyperphosphorylated tau protein in respiratory-related brainstem areas. These results have translational implications, since human tauopathy patients present unstable breathing during sleep, and therefore these rTg4510 mice could be a relevant model for studying the biological mechanisms involved in respiratory dysfunction due to tauopathy.

The other experimental work reported by Eugenín et al. is focused on the phenotypical effects of prolonged hypercapnia upon microglia in brainstem and hippocampus. The authors show that 30 min of 10% CO_2_ in air induces the transformation of homeostatic microglia into reactive microglia in specific brainstem respiratory nuclei of C57BL/6 and CF-1 mice, but not in Sp5 and hippocampal microglia. Immunofluorescence detection of surface inflammatory or regulatory markers to recognize functional states of reactive microglia, as well as ELISA detection of inflammatory (IL1β) and regulatory (TGFβ) cytokines by microglia, revealed that, likely, hypercapnia-induced microglia acquire an inflammatory phenotype. These results, also have translational implications given that the high levels of CO_2_ selected for the experiments mimics conditions of hypercapnia found in several human breathing disorders, such as Obesity Hypoventilation Syndrome (Shah et al., 2021), Congenital Central Hypoventilation Syndrome (Schafer et al., 1999; Lamon et al., 2012), and Sleep Apnea Hypopnea Syndrome (Wu et al., 2019). Whether microglia could play either a role in human hypoventilation syndromes or they simply exacerbate inflammatory damage in hypoventilation syndromes, are open questions.

Thus, this Research Topic of papers emphasizes new evidence that points to a more complex set of mechanisms for respiratory chemoreception than has been favored in the field in the past and indicates that much more work needs to be done to understand this vital function in health and disease.
